# Cell specific perfusion rates drive growth dynamics and metabolism in CHO N-1 perfusion processes independent of perfusion rate control method

**DOI:** 10.3389/fbioe.2025.1608889

**Published:** 2025-07-18

**Authors:** Julia Walther, Tiago Ribeiro da Costa, Lydia Winkler, Jochen Schaub, Tobias Habicher

**Affiliations:** Bioprocess Development Biologicals, Boehringer Ingelheim Pharma GmbH & Co. KG, Biberach an der Riß, Germany

**Keywords:** cell specific perfusion rate (CSPR), N-1 perfusion cultivations, specific metabolic rates, cell growth dynamics, medium exchange, CHO cell cultivation, process intensification

## Abstract

The production of monoclonal antibodies (mAbs) using Chinese Hamster Ovary (CHO) cell host systems often faces challenges in terms of manufacturing costs and efficiency. To address these challenges, process intensification with high seeding density production processes applying N-1 perfusion is utilized. This study delves into the impact of cell specific perfusion rate (CSPR) and the total exchanged medium in relation to the reactor working volume (iVVD) on cell growth dynamics and metabolic stability in N-1 perfusion cultivations. The effect of varying the perfusion rate increase (PRI) while keeping the amount of exchanged medium constant is investigated, revealing a positive correlation between iVVD and overall cell growth. However, this effect plateaus at higher values, indicating diminishing returns on cell growth with increased medium use. We found that CSPR directly influences the specific metabolic rates of several metabolites and amino acids, accelerating overall metabolism without necessarily affecting growth. Interestingly, the specific metabolic rates are driven by the CSPR after a metabolic adaptation until day 2.5. Besides adjusting perfusion rates every 24 h, the potential benefits of real-time CSPR control using a capacitance probe are explored. While real-time control offers more precise regulation of the perfusion rate, growth and metabolic behavior is comparable to predefined rates within the tested range. This study demonstrates that optimization of factors such as CSPR, iVVD, and PRI can lead to improved cell growth and viability with the potential to decrease media expenditure, thereby reducing manufacturing costs for the production of mAbs using CHO cell host systems.

## 1 Introduction

Monoclonal antibodies (mAbs) have become the best-selling drugs in the pharmaceutical market, with global annual sales projected to surpass $420 billion by 2029 (The Business Research Company, 2025). These complex proteins are typically expressed by Chinese Hamster Ovary (CHO) cell host systems in fed-batch mode with manufacturing bioreactors of up to 25 m^3^ (Kelley, 2009; Pohlscheidt et al., 2013). Despite the significant progress in mAb upstream processing, manufacturing costs remain substantially higher than those for small molecules (Xu et al., 2020a). A daily dose of a biologic costs 22 times more on average than that of a small molecule (Makurvet, 2021). This cost-pressure can be reduced by process intensification applying new manufacturing approaches and improved facility utilization strategies.

Upstream intensification strategies, such as N-1 perfusion, aim to outperform traditional fed-batch processes regarding volumetric productivity and resource utilization (Schaub et al., 2023). Despite its challenges (e.g., media consumption, operational complexity), especially at a large scale (Croughan et al., 2015; Schaub et al., 2023) N-1 perfusion can be implemented in existing facilities with minor modifications (Schaub et al., 2023; Stepper et al., 2020; Xu et al., 2020a). The implementation of N-1 perfusion aims to optimize cell growth by continuously eliminating waste and metabolic by-products, while simultaneously supplying fresh medium (Schulze et al., 2021). Cell retention, generally facilitated through a filter module, enhances the density of viable cells. This allows for the intensification of the fed-batch production stage, by providing high seeding cell densities (Müller et al., 2022; Padawer et al., 2013; Xu et al., 2020b; Yang et al., 2014). Thereby, the early growth and unproductive phase of fed-batch production is shifted to the N-1 culture step (Kloth et al., 2013), resulting in enhanced volumetric productivities and shorter process times (Xu et al., 2020b; Yang et al., 2016).

In a N-1 perfusion process the media exchange rate can be defined by the perfusion rate, expressed in vessel volumes of media per bioreactor volume per day and is usually increased over time, which enables the generation of more biomass (Bielser et al., 2018; Padawer et al., 2013; Wallocha and Popp, 2021). However, focusing solely on the perfusion rate without considering the viable cell density (VCD) does not allow for efficient and scalable processes (Konstantinov et al., 2006; Rittershaus et al., 2022). Thus, the cell specific perfusion rate (CSPR), the volume of perfused medium per cell and unit of time, is crucial for maintaining a constant nutrient supply throughout the process while removing growth-inhibiting by-products. However, high CSPRs increase the need for large media volumes, potentially creating high media production costs as well as logistical and operational difficulties in facilities (Karst et al., 2020; Konstantinov et al., 2006; Maria et al., 2023; Schulze et al., 2021).

Incorporating CSPR into perfusion rate control is feasible. A steady CSPR can be achieved by adjusting the perfusion rates according to set values and times, which aligns with the expected growth rate. However, this indirect method of control may result in inefficient medium use or nutrient deficiency in cultures where cell growth deviates from expectations and thus CSPR fluctuations occur (Brunner et al., 2021; Karst et al., 2020; Konstantinov et al., 2006; Pohlscheidt et al., 2013; Rittershaus et al., 2022). A more sophisticated strategy is real-time CSPR control, requiring online cell counts for automatic perfusion rate adjustment. This can be achieved using permittivity probes (Dowd et al., 2003). The VCD obtained allows for the regulation of the CSPR setpoint through feedback control. As a result, more constant cultivation conditions can be maintained (Müller et al., 2022; Rittershaus et al., 2022; Schulze et al., 2021; Warikoo et al., 2012; Xu et al., 2020b; Yang et al., 2014). This strategy aligns with the FDA’s Process Analytical Technology (PAT) guidelines, emphasizing real-time monitoring and control of critical process parameters (Bergin et al., 2022).

Most literature focuses on the intensified production bioreactor, merely demonstrating the successful implementation of N-1 perfusion at different scales and with various technologies (Brunner et al., 2021; Karst et al., 2016; Pohlscheidt et al., 2013; Stepper et al., 2020; Xu et al., 2020b). The impact of the perfusion rates and CSPR setpoints on cell growth is only briefly demonstrated in a few publications (Padawer et al., 2013; Yang et al., 2014), with deeper dives reported based on wet-lab experiments by Rittershaus et al. (2022) and through mechanistic modelling by Pogodaev et al. (2024). Nevertheless, these studies focus on perfusion rates to optimize cell growth and minimize medium expenditure, not analyzing the cellular metabolism. The influence of the perfusion rate and CSPR on specific metabolic rates has been mostly reported for N-stage perfusion cultivations (Dowd et al., 2003; Maria et al., 2023; Wolf et al., 2019). An investigation specific to N-1 perfusion has, to the best of our knowledge, only been reported by Schulze et al. (2021). However, this study only compares two different set-ups, one that maintains a constant CSPR of 0.05 nL/cell/day throughout cultivation and one that lowers the CSPR to 0.02 nL/cell/day for the last two cultivation days.

Our study provides a data set, in which different CSPR control strategies, CSPR setpoints and CSPR profiles were applied, resulting in an investigated CSPR range from 0.036 to 0.113 nL/cell/day. Additionally, the integral of the perfusion rate over the process time (iVVD) was varied between 3.8 and 12.0, and the PRI within a range of 1.4–2.0. This data was used to study the impact of the CSPR on both cell growth and cellular metabolism, providing the background necessary to develop efficient and scalable N-1 perfusion processes. Further, we compare the two aforementioned strategies of controlling the perfusion rates, addressing the question of whether a real-time CSPR control is necessary to optimize and implement efficient and scalable N-1 perfusion processes.

## 2 Materials and methods

### 2.1 Cell line, culture media and seed train

A Boehringer Ingelheim (BI) proprietary CHO-K1 glutamine synthetase (GS)^−/−^ cell line producing a recombinant protein was used in this study. The cells were cultivated in the BI proprietary chemically defined and animal component-free media. Cryovials containing the cell line were thawed and the cells were passaged in shake flasks (Corning, USA) of increasing size every second or third day. For lab-scale perfusion (3 L) four passages were performed. The shake flasks were cultivated in the Infors HT Multitron shaking incubator (Infors AG, Switzerland) at a temperature of 36.5°C, 5% CO_2_, 120 rpm (50 mm orbit) and without humidity control. The N-2 stage was performed in 8 L (Mavag AG, Switzerland) or 12 L (HWS Labortechnik, Germany) stirred bioreactors. The 3-day batch N-2 culture was maintained at a temperature of 36.5°C and a dissolved oxygen (DO) setpoint of 50%. The pH values were kept below the upper dead band of 7.20 through CO_2_ sparging.

### 2.2 N-1 perfusion culture

The N-1 perfusion culture was performed using a 3 L glass multifermenter system consisting of up to six autoclavable double-wall glass bioreactors (Applikon Biotechnology B.V., Netherlands). The bioreactors were equipped with a turbine agitator comprising a six-blade pitched-blade turbine above and a six-blade Rushton turbine below. A L-sparger with five 1 mm holes was used for aeration. The temperature was set at 36.5°C and a DO setpoint of 50% was maintained through sparging of process air and O_2_. Process air was sparged up until a threshold of 0.007 volumes gas per liquid volume per minute (vvm). This air flow was then held constant, as pure O_2_ was sparged to meet oxygen demands. The pH values were kept under the upper dead band of 7.20 through CO_2_ sparging. The culture was pumped through an external hollow-fiber filter S04-E65U-07 (Repligen GmbH, USA) at a constant recirculation rate of 0.5 L/min. The recirculation flow was controlled by the Levitronix console LCO-i100 (Levitronix GmbH, Switzerland), using the bearingless centrifugal pump PURALEV^®^ i30SU (Levitronix GmbH, Switzerland) and the clamp-on LFSC-i10X ultrasonic flow sensor (Levitronix GmbH, Switzerland). Perfusion rates were controlled using a feedback control based on scales (PBK989-AB30, Mettler-Toledo Inc., USA), in which both the reactor and the permeate collection container were placed on a scale. Glucose bolus additions were provided for glucose concentrations under 3 g/L. The cultivation was conducted for 6 days, and perfusion was initiated 24 h after inoculation.

The perfusion rate was controlled utilizing two different methods. With the first approach, the perfusion rate was adjusted daily after sampling, according to pre-defined perfusion rates. The perfusion rates were held constant during 24-h intervals. This strategy was defined under the term fixed working reactor volumes in volumes per day (VVDs). The second method, the automatic real-time control of the CSPR, required permittivity probes (Incyte Arc, Hamilton Company, USA) to monitor the VCD. The calculation of the VCD from the measured permittivity was performed based on a linear regression between the permittivity and the VCD (Supplementary Appendix Figure 4). The implemented control loop regulated the CSPR by adjusting the perfusion rate every 30 min, utilizing the mean online VCD over this specific time interval. This method was defined under the term real-time CSPR control.

### 2.3 Analytical methods

Daily sampling was performed throughout the cultivations. Cell counts and viabilities were determined using either the CEDEX Analyzer (Roche Diagnostics, Germany) or the ViCELL-BLU Cell Counter (Beckman Coulter, USA). Supplementary Appendix Figure 5 provides a comparison between the measurements of both devices for the same cultivation. Accumax™ (Innovative Cell Technologies, USA), a cell detachment solution, was used for N-1 perfusion samples. Glucose, lactate, and ammonia concentrations were measured using the Konelab™ Prime 60i system (Thermo Fisher Scientific Inc., USA). Amino acid analysis was conducted using the Agilent 6890N network gas chromatograph (Agilent Technologies INC., USA) with the KG0-7165 EZ:faast™ analysis kit (Phenomenex Inc., USA). A list of the evaluated amino acids and corresponding abbreviations: ALA: Alanine; ASN: Asparagine; ASP: Aspartate; GLN: Glutamine; GLU: Glutamate; GLY: Glycine; HIS: Histidine; ILE: Isoleucine; LEU: Leucine; MET: Methionine; PHE: Phenylalanine; PRO: Proline; SER: Serine; THR: Threonine; TRP: Tryptophan; TYR: Tyrosine; VAL: Valine.

### 2.4 Principal component analysis (PCA) and other statistical methods

A PCA was performed on a dataset consisting of the daily specific growth rate and daily specific metabolic rate of glucose, lactate and the 17 amino acids (ALA, ASN, ASP, GLN, MET, PHE, PRO, SER, GLU, GLY, HIS, ILE, LEU, THR, TRP, TYR, VAL). The PCA loadings of the first two principal components of the PCA depicted in Figure 2 are shown in Supplementary Appendix Figure 1. Ammonia was not included, as data was not available for all cultivations. The dataset was centered prior to performing the PCA by dividing each value by the highest value observed for the respective variable. By doing so, the highest value for each variable was set to 1, while maintaining the relative proportions between the different data points. The MATLAB R2023a function “pca” in combination with the ALS algorithm was used (The MathWorks, Inc., Natick, Massachusetts, United States). This algorithm was chosen due to the presence of missing values resulting from the removal of individual outliers (on average 5.6% outliers were identified over all investigated metabolites). Outliers were identified as instances of negative rates that were inconsistent with the overall dataset context.

Pearson correlation coefficients were calculated to assess the linear relationships between the daily specific growth rate and the daily specific metabolic rates of glucose, lactate, and the 17 amino acids. The MATLAB R2023a function “corr” was used for this analysis (The MathWorks, Inc., Natick, Massachusetts, United States). This function computes the Pearson correlation coefficient, which measures the strength and direction of the linear relationship between two variables.

Additionally, linear regression analysis was performed to model the relationship between the daily specific growth rate and the daily specific metabolic rates of glucose, lactate, and the 17 amino acids. The MATLAB R2023a function “fitlm” was utilized for this purpose (The MathWorks, Inc., Natick, Massachusetts, United States). This function fits a linear model to the data, providing estimates of the regression coefficients, confidence intervals, and various goodness-of-fit statistics.

### 2.5 Perfusion specific parameter calculations

A fundamental perfusion specific parameter is the perfusion rate, which can be expressed in terms of the VVD. The total exchanged medium in relation to the reactor working volume can be expressed as the integral of the perfusion rate over the process time (iVVD).

In this study, each cultivation was dissected into daily intervals, for which the mean cell count ${VCD}_{mean}$ [10^6^ cells/mL], specific growth rate µ [1/day] and CSPR [nL/cell/day] were calculated according to Equations 1–3, respectively:

$${VCD}_{mean}=\frac{{VCD}_{i-1}+{VCD}_{i}}{2}$$ (1)

$$µ=\frac{\ln\left({VCD}_{i}/{VCD}_{i-1}\right)}{\left(t_{i}-t_{i-1}\right)}$$ (2)

$$CSPR=\frac{VVD}{{VCD}_{mean}}$$ (3)

Where VCD [10^6^ cells/mL] is the measured viable cell density and t [day] represents the process time. The indices i−1 and i refer to the timepoint of each parameter. The VVD [L/L/day] is the perfusion rate set for the time interval.

The daily consumption or production of each metabolite ${met}_{cons/prod}$ [mmol] was calculated according to Equation 4, from which the cell specific consumption rate $\dot{q}$ [pmol/cell/day] was calculated, as described in Equation 5:

$${met}_{cons/prod}=V\times \left(\left(c_{\;i-1}-c_{\;i}\right)+\left(t_{i}-t_{i-1}\right)\times VVD\times \left(c_{\;in}-\frac{c_{i-1}+c_{i}}{2}\right)\;\right)$$ (4)

$$\dot{q}=\frac{{met}_{cons/prod}}{\left(t_{i}-t_{i-1}\right)\times {VCD}_{mean}\times V}$$ (5)

where V [L] is the bioreactor working volume and c [mmol/L] the concentration of the metabolite. The indices i−1 and i refer to the timepoint of each parameter. in refers to incoming medium. For the calculation of the daily consumption of glucose, the amount of glucose [mmol] added during bolus additions must be considered. Due to the nature of Equation 5, a positive value of $\dot{q}$ indicates a consumption of the metabolite, while a negative value indicates its production.

To maintain a consistent evaluation method, the same principles as described in Equations 1–5 were applied to analyze cultivations using a real-time CSPR control. Therefore, only VCDs measured by the cell count devices were considered. Given the constant change in perfusion rate in real-time CSPR controlled experiments, the daily perfusion rate was calculated using the average of the rates at the start and end of the daily interval. This ensures a fairer comparison of CSPR values between processes with pre-defined perfusion rates and real-time CSPR control.

## 3 Results

### 3.1 The impact of the amount of exchanged media

A straightforward method to vary the CSPR while maintaining a constant seeding cell density (SCD) is to adjust the amount of exchanged media. Therefore, the iVVD values were varied in between 6.0, 7.5 and 9.0. Perfusion rates were pre-defined with a daily perfusion rate increase (PRI) of 2.0, meaning that the perfusion rates were doubled daily. This PRI factor was chosen under the assumption of a cell count doubling time of 24 h and a constant specific growth rate during the perfusion cultivation.

**FIGURE 1:**
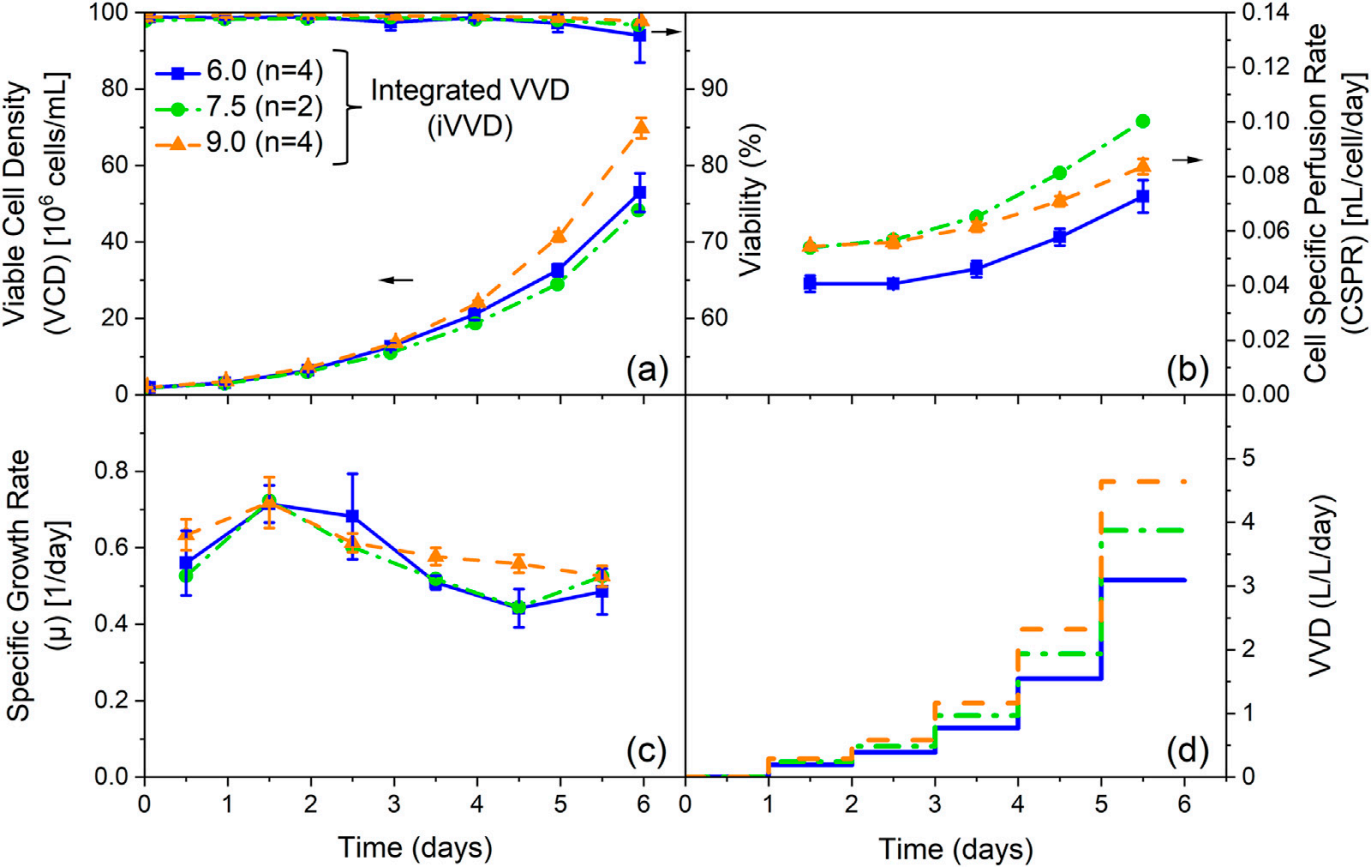
Influence of the integrated VVD (iVVD) with perfusion rate increase (PRI) of 2.0 on process performance and cell specific perfusion rate (CSPR). **(a)** Viable cell density (VCD) and viability, **(b)** CSPR, **(c)** specific growth rate (µ) and **(d)** pre-defined stepwise VVD profiles with a daily PRI of 2.0 over process time. The iVVD is the sum of the daily VVDs. n = number of cultivations. Mean values are depicted for n ≥ 2. Standard deviations are depicted by error bars for n ≥ 3.

**FIGURE 2:**
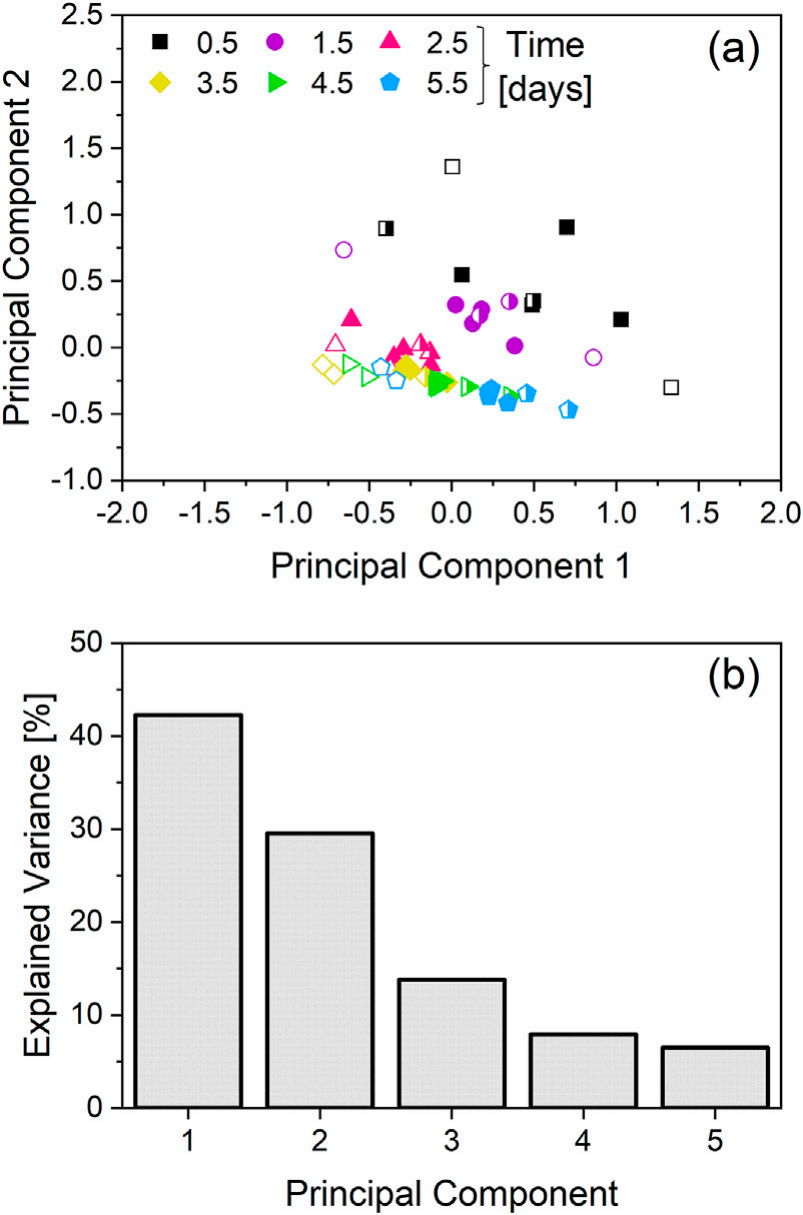
Principal component analysis (PCA) of the specific growth rate and specific metabolic rates. The cultivations depicted in Figure 1 are the source of the specific rates used for the PCA. Two cultivations with an integrated VVD (iVVD) of 6.0 were not included in the dataset, due to lacking amino-acid data. While the cultivations with an iVVD of 9.0 were included in the dataset, ammonia data was not included due to unavailability for all cultivations. **(a)** PCA score plot. Datapoints colored and shaped according to the corresponding timepoint. Open symbols correspond to cultivations with an iVVD of 6.0, half-filled symbols correspond to cultivations with an iVVD of 7.5 and solid symbols to cultivations with an iVVD of 9.0. **(b)** Explained variance of the first 5 principal components.

Figure 1a depicts the VCD and viability profiles for these cultivations. Final VCDs of 48 · 10^6^ and 53 · 10^6^ cells/mL were achieved for the cultivations with an iVVD of 7.5 and 6.0, respectively. Cultivations with an iVVD of 9.0 reached final VCDs of 70 · 10^6^ cells/mL. Viabilities were high for all cultivations (>97%), though higher viabilities on the final day can be seen with increasing iVVD. The specific growth rate profiles are similar for all cultivations. An initial growth rate of 0.52–0.63 1/day at day 0.5 increased up to 0.72 1/day in the subsequent 24 h. The specific growth rate then dropped on day 3.5, remaining at this lower level until the end of the cultivation (Figure 1c). The drop in the specific growth rate is especially visible in the cultivations with an iVVD of 6.0 and 7.5. The combination of a decreasing specific growth rate and a constant perfusion rate increase (Figure 1d) is reflected in the CSPR profiles (covering a range of 0.04–0.11 nL/cell/day), which trend upward from day 3.5 onward (Figure 1b), increasing up to 85% over the cultivation time (for iVVD of 6.0). Additional data on the impact of varying iVVD values (4.5, 6.0, and 10.5) and a constant PRI of 2.0 of a different clone of the CHO-K1 GS cell line producing a distinct product showing the same trends on VCD, viability, CSPR, and specific growth rates are provided in Supplementary Appendix Figures 7a–d. To further explore these cultivations, a Principal Component Analysis (PCA) of the specific rates was performed (Figure 2).

The PCA scores plot shows two distinct clusters. The first cluster corresponds to datapoints collected on day 0.5 and 1.5, and the second cluster corresponds to datapoints from day 2.5 onwards. The cluster which exhibits higher scattering consists of datapoints collected on day 0.5 and 1.5, whereas datapoints from day 2.5 onwards cluster more tightly (Figure 2a). The explained variance of the first two principal components (PC) exceeds 70%, with the PC1 comprising 42% and PC2 comprising 30% of the explained variance (Figure 2b). To identify the specific metabolic rates that are driving the observed differences in the metabolic states of the cell culture (Figure 2), the loadings of the first two principal components are plotted in Supplementary Appendix Figure 1. For PC1, ASP, GLU and TYR, PHE show the two highest absolute values in the positive and negative directions, respectively. For PC2, ASP and THR, PRO are the main factors driving the observed differences in the positive and negative directions, respectively.

**FIGURE 3:**
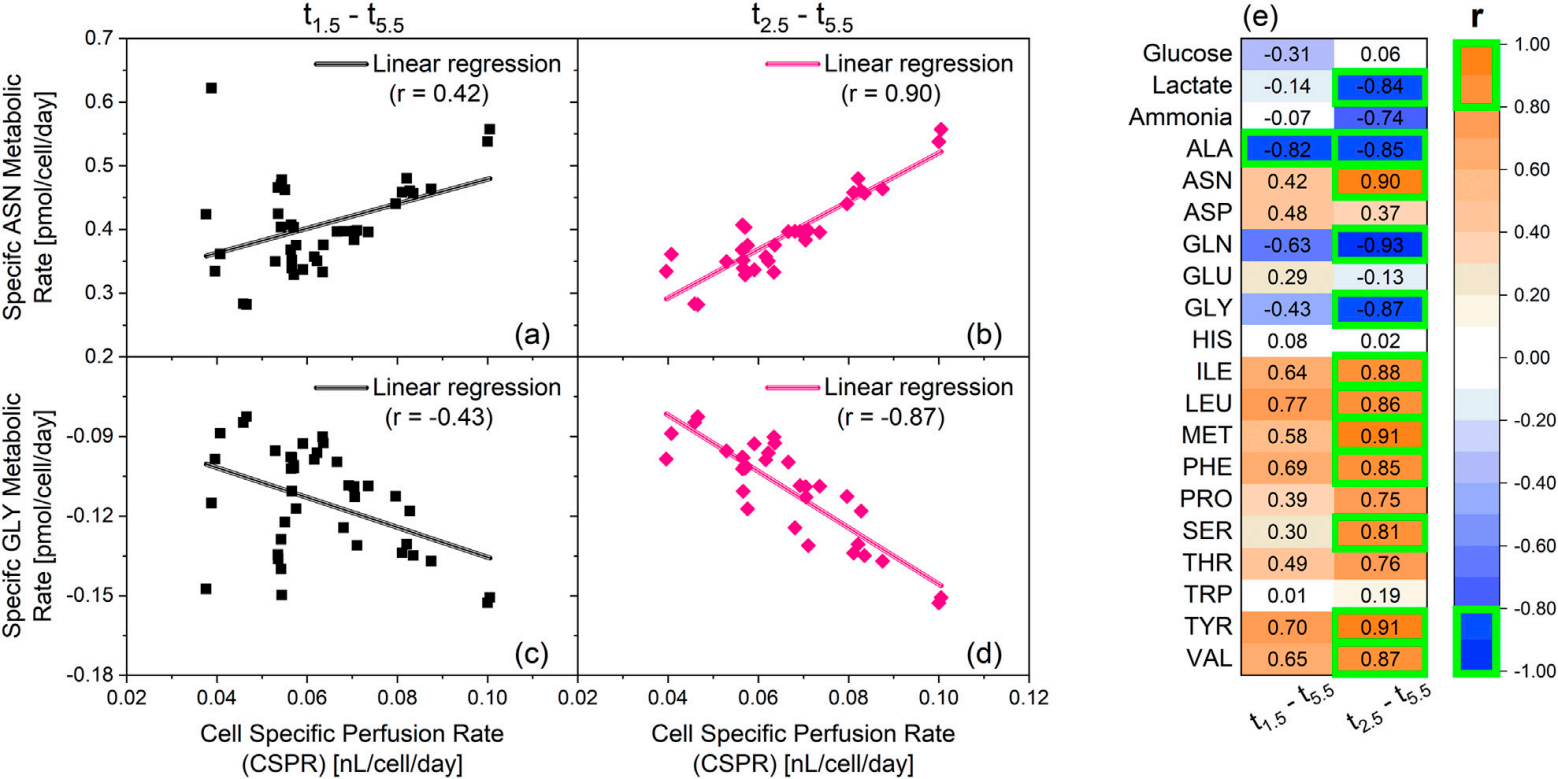
Linear correlation analysis between specific metabolic rates and cell specific perfusion rates (CSPR). The cultivations depicted in Figure 1 are the source of the specific rates used for the correlation analysis. Two cultivations with an integrated VVD (iVVD) of 6.0 were not included in the dataset, due to lack of amino-acid data. Ammonia data was not available for cultivation with an iVVD of 9.0. Shown in this figure: Specific metabolic rate of asparagine (ASN) between days 1.5–5.5 **(a)** and day 2.5–5.5 **(b)** over CSPR. Specific metabolic rate of glycine (GLY) between day 1.5–5.5 **(c)** and day 2.5–5.5 **(d)** over the CSPR. Positive metabolic rates indicate a consumption of the metabolite, while negative metabolic rates indicate a production of the metabolite. Linear regressions and the corresponding Pearson correlation coefficients (r) are provided. **(e)** List of the Pearson correlation coefficients of the linear correlations between the specific metabolic rates and CSPR from day 1.5–5.5 and from day 2.5–5.5. r > 0 indicates a consumption of the metabolite. r < 0 indicates a production of the metabolite. |r| ≥ 0.8 are highlighted by a green box.

To better understand the effect of the CSPR on the metabolism of the culture depicted as clusters in Figure 2a, the daily CSPR was plotted against the daily specific metabolic rates (Figure 3). Datapoints from day 0.5 are not included, as perfusion mode was only initiated on day 1.

Data from day 1.5 until day 5.5 show absolute Pearson correlation coefficients (|r|) below 0.8, with alanine (ALA) being the only exception (r = −0.82) (Figure 3e). Overall, the absolute Pearson correlation coefficients averaged at |r| = 0.44 between the analyzed metabolites. This is exemplarily depicted for asparagine (ASN) and glycine (GLY) in Figures 3a,c, respectively. After removing the datapoints from day 1.5, a drastic increase in |r| was observed for asparagine and glycine (Figures 3b,d). This trend is also visible for most metabolites, with 12 from 20 showing |r| ≥ 0.8 after removing data from day 1.5 (Figure 3e). This implies that the CSPR has little influence on the metabolic rates of day 1.5. However, from day 2.5 onward, an increase in CSPR correlates with an increase in the production (r < 0) or consumption (r > 0) of several metabolites. In fact, only glucose, aspartate (ASP), glutamate (GLU), histidine (HIS) and tryptophan (TRP) showed low correlation coefficients from day 2.5 onward, averaging at |r| = 0.15. The specific glucose and lactate metabolic rates as a function of CSPR for varying iVVD values (4.5, 6.0, and 10.5) are presented in Supplementary Appendix Figures 7e–f conducted with a different clone of the CHO-K1 GS cell line producing a distinct product showing the same linear relationship between CSPR and the investigated specific metabolic rates.

### 3.2 CSPR profiles at constant iVVD

**FIGURE 4:**
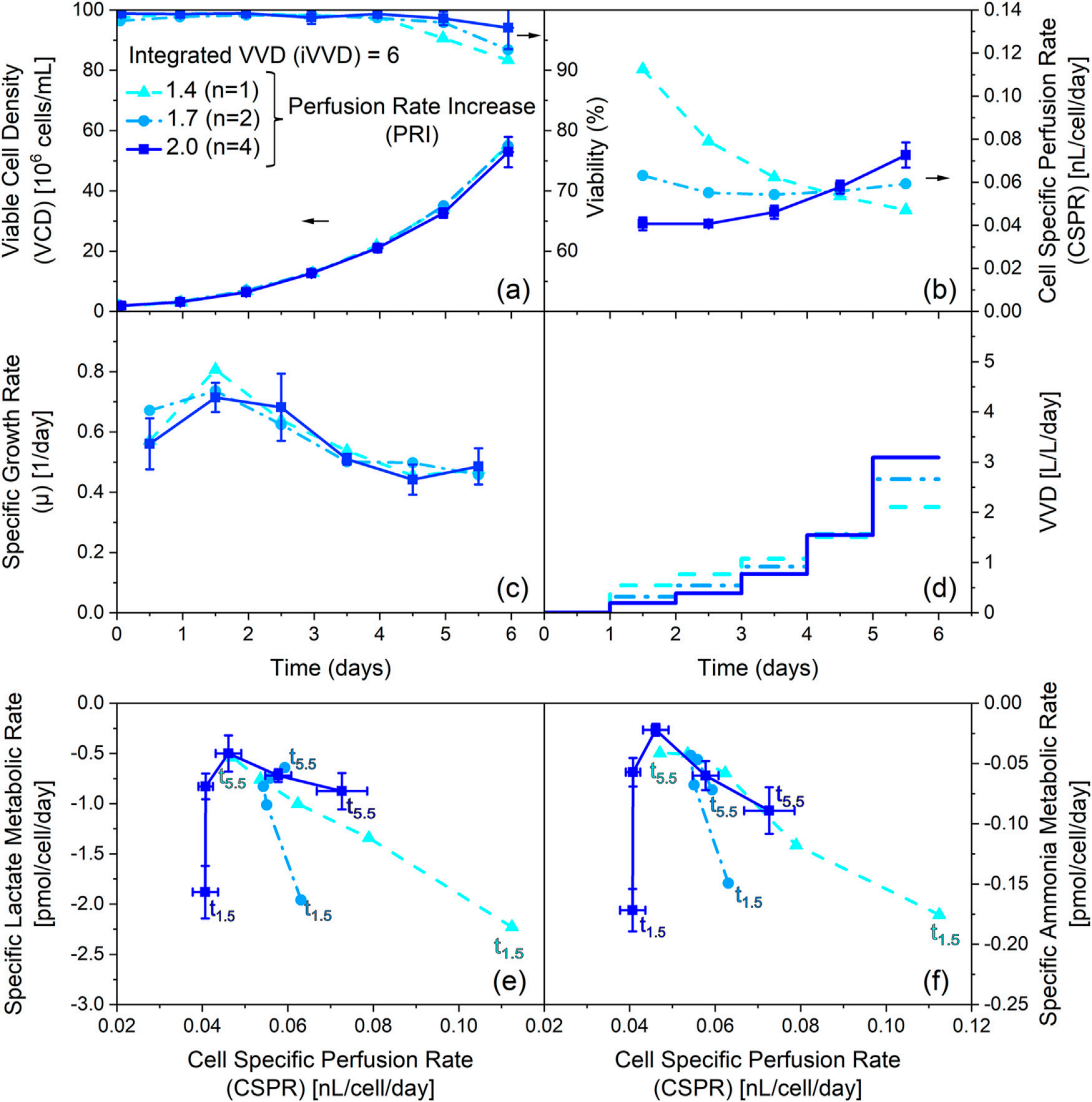
Influence of varying the perfusion rate increase (PRI) at constant integrated VVD (iVVD) on process performance, cell specific perfusion rate (CSPR) and specific metabolic rate of lactate and ammonium. **(a)** Viable cell density (VCD) and viability, **(b)** CSPR, **(c)** specific growth rate (µ) and **(d)** pre-defined stepwise VVD profiles with daily PRIs of 1.4, 1.7 and 2.0 over process time. The iVVD is the sum of the daily VVDs. **(e)** Specific lactate metabolic rate, **(f)** specific ammonia metabolic rate profiles over CSPR. Negative metabolic rates indicate a production of the metabolite. Datapoints labelled with t_1.5_ refer to the specific metabolic rates on day 1.5. Datapoints labelled with t_5.5_ refer to the specific metabolic rates on day 5.5. Datapoints in between correspond to the remaining days and are connected chronologically. n = number of cultivations. Mean values are depicted for n ≥ 2. Standard deviations are depicted by error bars for n ≥ 3.

The previous chapter focused on the impact of the iVVD in N-1 perfusion cultivations. However, the effect of the perfusion rate increase (PRI) was not investigated. In this section, the PRI was varied while the amount of exchanged medium was kept constant. Figure 4 portrays the results obtained by varying the PRI between 1.4, 1.7 and 2.0 while maintaining an iVVD of 6.0.

As depicted in Figure 4a all cultivations reached similar final VCDs between 53 · 10^6^ and 55 · 10^6^ cells/mL, despite different PRI values. The similarity of the VCD profiles in combination with a large variation in PRI resulted in different CSPR profiles (Figure 4b). A PRI of 1.4 resulted in a decrease of the CSPR from 0.11 to 0.05 nL/cell/day over the cultivation time, while a PRI of 1.7 led to a constant CSPR of 0.06 nL/cell/day throughout the cultivation. A PRI of 2.0 led to an increase in CSPR over the cultivation time from 0.04 to 0.07 nL/cell/day. Nevertheless, these profiles show no impact on the specific growth rate profiles, which are very comparable between all cultivations (Figure 4c). However, the viability profiles indicate a slight trend, showing a lower drop in the viability on day 6 for increasing CSPR profiles (Figure 4a).

Figures 4e,f depict the specific metabolic rates of lactate and ammonia for these cultivations. The specific metabolic rates are comparable on day 1.5, despite the significant variation in CSPR (0.04–0.11 nL/cell/day). However, subsequent datapoints reveal notable differences in the metabolic state based on the PRI settings. Specifically, cultivations with a PRI of 2.0 show an increase in specific production rates over time (see also Supplementary Appendix Figures 7e, f), whereas cultivations with a PRI of 1.7 exhibit relatively constant specific rates of metabolite production. In contrast, the cultivation with a PRI of 1.4 depicts a decline in specific production rates of lactate and ammonia throughout the cultivation. These datapoints establish a linear correlation between the CSPR and the specific metabolic rates of lactate and ammonia, similar to Figures 3a–d, which depict the same trend for asparagine and glycine. However, Figures 4e,f demonstrate that these linear correlations are independent of the cultivation time (see also Supplementary Appendix Figures 7e, f). Moreover, they underscore the negligible influence of the CSPR on cellular metabolism on day 1.5.

Overall, these findings highlight the importance of the PRI on cellular metabolism and emphasize the role of the CSPR in determining the metabolic state of the cells. However, the data also indicate that the PRI and the different CSPR profiles it creates, have no significant effect on cell growth at constant iVVD.

### 3.3 Real-time CSPR control

**FIGURE 5:**
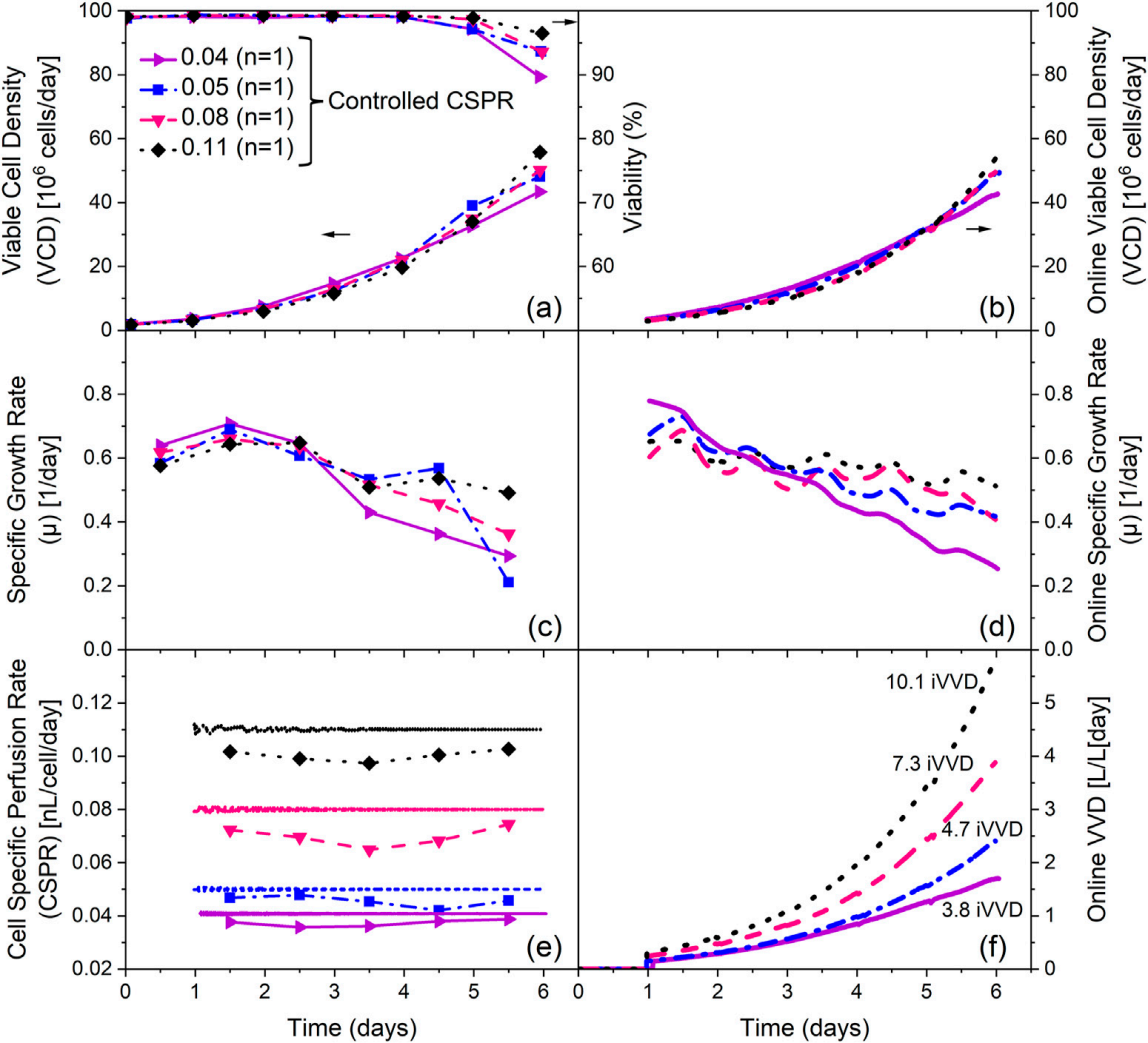
Influence of the cell specific perfusion rate (CSPR) on process performance using real-time CSPR control. **(a)** Viable cell density (VCD) and viability, **(b)** online VCD, **(c)** specific growth rate (µ) calculated with offline VCD data, **(d)** specific growth rate (µ) calculated with online VCD data (Lowess smoothing, Span = 0.2), **(e)** CSPR calculated with offline and online VCD data and **(f)** online VVD profiles over process time. The integrated VVD (iVVD) are provided in **(f)** Lines with symbols represent data based on offline VCD measurements. Lines without symbols represent online data based on measurements from permittivity probes (calibration curve provided in Supplementary Appendix Figure 4). n = number of cultivations.

N-1 perfusion processes utilizing an automatic, real-time CSPR control were performed. Each cultivation had a specific constant CSPR setpoint, ranging from 0.04 to 0.11 nL/cell/day. Both online and offline data are shown in Figure 5.

Offline cell counts and viabilities indicate a positive impact on both parameters with increasing CSPR. The highest final VCD of 56 · 10^6^ cells/mL was reached in the cultivation with the highest CSPR setpoint, while the lowest final VCD of 43 · 10^6^ cells/mL was reached in the cultivation with the lowest CSPR setpoint. This cultivation also shows the largest drop in viability between day 5 and 6, from 97% to 90% (Figure 5a). The offline specific growth rate (µ) profiles also show a positive impact of higher CSPR setpoints, as illustrated by a lower decline in µ over the course of the cultivation (Figure 5c). These findings are consistent with the online data obtained from the permittivity probes (Figures 5b,d).

The online CSPR profiles show minimal oscillations from the respective setpoint, as shown in Figure 5e. However, the offline CSPR values calculated with the offline cell counts are, on average, 10% lower than the setpoint. The perfusion rate profiles required to maintain the CSPR setpoints are depicted in Figure 5f, along with the resulting iVVDs which range from 3.8 (0.04 CSPR) to 10.1 (0.11 CSPR).

### 3.4 Specific growth and metabolic rates

**FIGURE 6:**
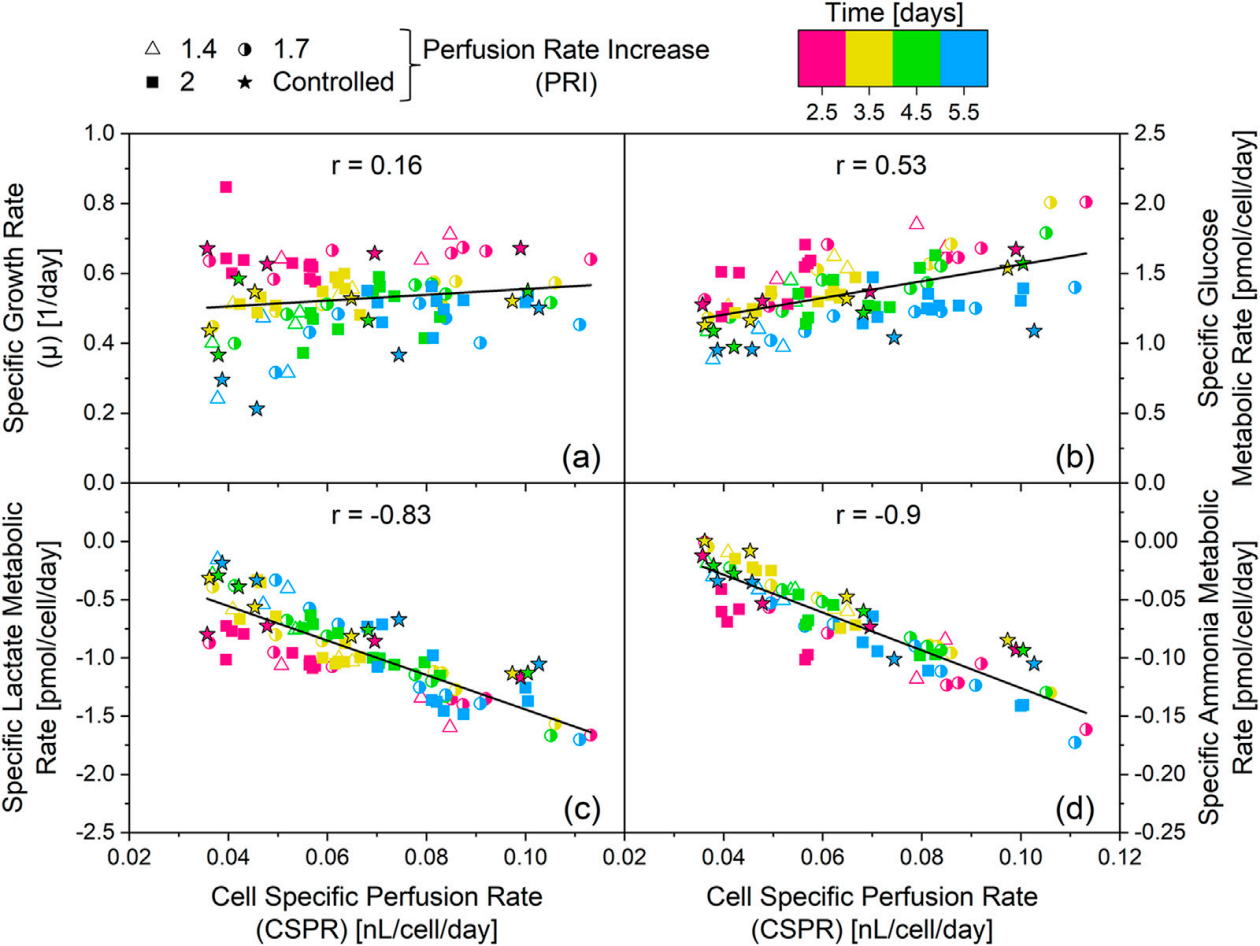
Influence of the cell specific perfusion rate (CSPR) on the specific growth rate **(a)** and the specific metabolic rates of glucose **(b)** lactate **(c)** and ammonia **(d)** The cultivations depicted in Figures 1, 5, and Supplementary Appendix Figures 2, 3 are the source of the specific rates. The resulting linear regressions are depicted by the black lines and the value of the corresponding Pearson correlation coefficient (r) is provided. The corresponding R^2^ are as follows: **(a)** R^2^ = 0.03, **(b)** R^2^ = 0.29, **(c)** R^2^ = 0.68, **(d)** R^2^ = 0.81. Positive metabolic rates indicate a consumption of the metabolite, while negative metabolic rates indicate a production of the metabolite.

The following chapter provides an overview on the specific growth rate and the specific metabolic rates of glucose, lactate and ammonia for all cultivations performed within this work. This dataset includes real-time CSPR controlled cultivations and fixed daily VVD cultivations with different iVVDs and PRIs, providing a wide spectrum of CSPRs (0.035–0.113 nL/cell/day) and different CSPR profiles. Figure 6 depicts the relationship between the CSPR and different cell specific rates.

The Pearson correlation coefficient (r) = 0.16 of the linear regression between the CSPR and the specific growth rate (Figure 6a) reveals no correlation between the two parameters. Moreover, no significant correlation (|r| < 0.8) was observed between the CSPR and the specific metabolic rate of glucose (Figure 6b). However, |r| ≥ 0.8 were found for the specific metabolic rates of ammonia and lactate (Figures 6c,d). This strong correlation was obtained independent of the culture day, with a dataset encompassing all performed cultivations, from fixed VVD cultivations with different PRI and iVVD, to real-time CSPR controlled cultivations with different CSPR setpoints. This indicates that the CSPR directly affects the specific metabolic rates of these metabolites (see also Supplementary Appendix Figures 7e, f).

**FIGURE 7:**
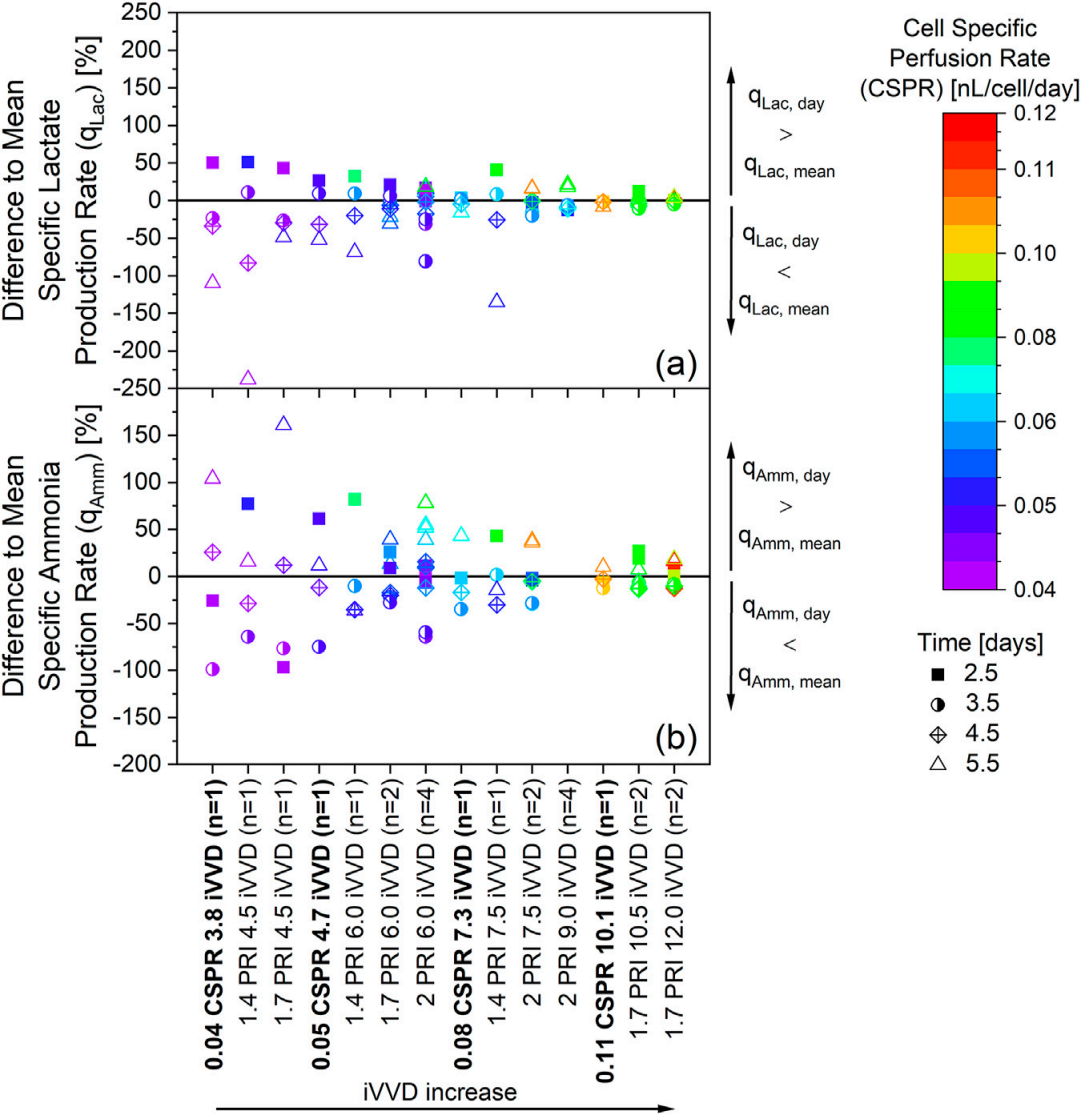
Fluctuation of the specific metabolic rates within real-time controlled cell specific perfusion rate (CSPR) in bold and fixed daily VVD cultivations. The cultivations depicted in Figures 1, 5, and Supplementary Appendix Figures 2, 3 are the source of the specific rates. Ammonia data was not available for cultivations with an iVVD of 9.0. Percentual difference between the daily and mean specific production rate of lactate **(a)** and ammonia **(b)** The mean specific production rates q_Lac, mean_ and q_Amm, mean_ (day 2.5 to day 5.5) of each individual cultivation were defined as zero. The different cultivations along the x-axis are ordered from left to right according to increasing iVVD, ranging from 3.8 to 12.0. A narrow distribution of datapoints along the y-axis represents a constant specific metabolic rate over time. A change in colors for a particular cultivation represents a change of the CSPR over time. n = number of cultivations.

To investigate whether a real-time CSPR control leads to a more constant metabolic state compared to fixed daily VVDs, the fluctuation of the daily specific metabolic rates relative to the cultivations mean metabolic rate was calculated. This analysis, which is visualized in Figure 7, was performed for datapoints from day 2.5 onward, as these were previously shown to correlate with the CSPR (Figure 3).

The fluctuation in the specific metabolic rates of both lactate (Figure 7a) and ammonia (Figure 7b) show a similar pattern. At low iVVDs, where the CSPRs do not exceed 0.05 nL/cell/day, the datapoints are highly scattered along the y-axis. With increasing iVVD, the fluctuation of the specific metabolic rates of lactate and ammonia decreases, indicating a more constant metabolism throughout the cultivation. This general trend is visible independent of the perfusion control strategy, be it fixed daily VVDs or real-time CSPR control. Nevertheless, the data also reveal a lower fluctuation along the y-axis in cultivations with more constant CSPR profiles, as is visible in fixed VVD cultivations with a PRI of 1.7 (Figure 4) and in real-time CSPR control cultivations. The specific metabolic rates of ammonia, for example, range between +40% and −30% from the q_Amm, mean_ in these cultivations, while those with a PRI of 1.4 range from +80% to −40% and those with a PRI of 2.0 range from +80% and −65% (Figure 7b). As can be seen by the color of the datapoints in Figure 7 and the CSPR profiles in Figure 4b, a PRI of 1.7 provided a constant CSPR, unlike a PRI of 1.4 or 2.0. A further example of the effect of the CSPR on the fluctuations in the specific metabolic rates is visible when comparing the cultivation with a real-time CSPR control at 0.08 nL/cell/day with the fixed VVD cultivation with a PRI of 1.4 and iVVD of 7.5. Despite having a similar iVVD, fluctuations in the specific lactate production rate are minimal for the cultivation with real-time CSPR control, while the q_Lac_ for the cultivation with a PRI of 1.4 ranges between +50%, on day 2.5, and −135%, on day 5.5.

## 4 Discussion

### 4.1 N-1 perfusion performance–impact of CSPR on cell growth dynamics

Cellular growth dynamics are influenced by different iVVDs and CSPR profiles. Cultivations reveal a positive correlation between the iVVD and overall cell growth, leading to an increase in the final VCD of N-1 perfusion cultivations (Figure 1; Supplementary Appendix Figure 2, Supplementary Appendix Figure 7). A higher iVVD provides more nutrients for cell growth while simultaneously removing waste products, thereby promoting cell growth. A higher iVVD usually results in a higher CSPR (Supplementary Appendix Figure 2b). However, this increased iVVD can also lead to higher growth rates, which, despite resulting in higher final VCDs, may reduce the CSPR (see Figure 1 iVVD 7.5 and 9; Supplementary Appendix Figure 2; Supplementary Appendix Figure 7). This trend is supported by the data in Supplementary Appendix Figure 7 for a different clone of the CHO-K1 GS cell line producing a distinct product, which demonstrate the impact of varying iVVD values (4.5, 6.0, and 10.5) at a constant PRI of 2.0 on VCD, viability, CSPR, and specific growth rates, highlighting the reproducibility of these trends. It also aligns with previous findings (Dowd et al., 2003; Padawer et al., 2013; Pogodaev et al., 2024; Pohlscheidt et al., 2013; Rittershaus et al., 2022; Yang et al., 2014). Despite the variations in iVVD, the specific growth rate profiles remained rather constant until day 2.5 for all tested setups. From this point onward, a more pronounced decrease in the daily specific growth rate was observed in processes with lower iVVD (Supplementary Appendix Figure 2 and Supplementary Appendix Figure 7). However, none of the tested set-ups resulted in a constant growth rate over time, a finding consistent with the reports of Pohlscheidt *et al.* and Stepper *et al.* (Pohlscheidt et al., 2013; Stepper et al., 2020).

The data suggest a non-linear relationship between increasing iVVD and final VCDs, indicating diminishing returns on cell growth with increased medium use (Supplementary Appendix Figure 6). Our data reveal, despite the lack of correlation of the CSPR with the daily specific growth rate (Supplementary Appendix Figure 6) that the mean growth rate increased with increasing iVVD but might plateau at some point outside of the evaluated range (Supplementary Appendix Figure 6). We confirmed this trend for two different clones of the CHO-K1 GS cell line producing a distinct product, underscoring the robustness of the observed relationship. This suggested plateau, which is also reported by Pogodaev (Pogodaev et al., 2024) based on mechanistic models aimed at reducing media expenditures while preserving cellular performance, is best exemplified by comparing the iVVD between the cultivation with the lowest and highest final VCD. The cultivation with the lowest final VCD (43 · 10^6^ cells/mL) required 3.8 iVVD of perfusion media, while the highest final VCD (70 · 10^6^ cells/mL) was reached in a cultivation with 10.5 iVVD of perfusion media. This represents an increase of 63% in the final VCD for a 176% increase in medium expenditure. It implies that it is possible to reduce medium consumption in the pre-stage while maintaining the final VCD within the targeted range, which is particularly important for efficient cell cultivation in large-scale applications. Identifying the precise inflection point where increased iVVD no longer yields proportional gains in final VCD would require a detailed cost-of-goods (COG) analysis that accounts for the production stage and return on investment, which is primarily driven by the final product concentration. This underscores the importance of integrating economic considerations into process optimization, particularly for large-scale applications where media expenditures significantly impact overall production costs.

When iVVD is kept constant, as depicted in Figure 4, and only the PRI and thus CSPR profiles are varied, it allows for the investigation of the distinct influence of these variables on growth performance. Interestingly, despite different PRI values, no significant difference in growth rate or VCDs were observed indicating robust cell growth within the tested CSPR range and profiles. This aligns with Pogodaev’s mechanistic modelling approach. However, beyond Pogodaev’s findings, our data show that the CSPR does affect viability and metabolic activity. A decrease of CSPR over process time (PRI of 1.4) results in a decline in viability over the last 2 days of perfusion, while an increase in CSPR with a PRI of 2 (Figure 4B) leads to improved viability (Figure 4A). The trend of a decreasing viability with a decreasing CSPR over process time is also visible for cultivations with a PRI of 1.4 but varied iVVDs (Supplementary Appendix Figure 3). Comparing the influence of the CSPR profile in Figure 1 (PRI 2; iVVD 7.5) to the same settings but reduced PRI of Supplementary Appendix Figure 3 (PRI 1.4, iVVD 7.5), viability is reduced from 98% to 89% at process end, respectively. The same trend of CSPR on viability can be observed for metabolic rates of lactate and ammonia (Figures 4e,f). Metabolic activity increases after 1.5 days of perfusion with an increasing CSPR, independent of process time. These findings suggest that the effect of CSPR becomes visible towards the end of cultivation time, implying that overall media consumption can be reduced while productivity, defined as the number of cells per unit of consumed medium, can be increased. This is particularly interesting for scale up and offers potential for further optimization strategies of perfusion processes. Additionally, the impact of increased metabolic activity observed in our data, needs to be investigated during the production stage.

### 4.2 N-1 perfusion cultivation - CSPR control and metabolic stability

The real-time control of CSPR provides the advantage of continuous monitoring and control of this process parameter. In this study, we briefly explored, the comparison between this approach and predefined rates, particularly focusing on the lowest CSPR, where regulation was hypothesized to have the most significant impact. Our data shows good comparability between the two methods, suggesting that precise real-time control of CSPR may not necessarily enhance cell proliferation (Figure 5; Supplementary Appendix Figure 2). Additionally, the metabolic rate remains consistent at the same CSPR, whether it's controlled in real-time or predefined (Figure 6). This indicates that the consistency of CSPR is directly linked to the consistency of metabolism.

Both offline and online data for VCD, viability, and growth rate align well with the online data. However, the offline CSPR values, calculated using offline cell counts, were found to be approximately 10% lower than the setpoint on average. The accuracy of these measurements is dependent on the robustness of the prior calibration, with any deviation potentially causing discrepancies in CSPR setpoint calculations. Despite more extensive calibration data, a persistent discrepancy remains between offline VCD values and controlled CSPR setpoints due to the intrinsic inaccuracy of offline measurements. It is important to note that the observed offset to the setpoint can be accounted for by these calibration deviations (Supplementary Appendix Figure 4). Despite these deviations, no significant signal drift was observed during the cultivations (see Supplementary Appendix Figure 3). To further contextualize the calibration accuracy and potential error propagation in VCD estimation, we refer to the review by Bergin et al. (2022), which highlights that the accuracy of bio-capacitance-based cell density measurements is inherently dependent on the quality of the calibration model and the specific cell line and culture conditions used (Bergin et al., 2022). The authors emphasize that while bio-capacitance offers high sensitivity to viable biomass, it is also susceptible to variations in media conductivity and cell morphology, which can introduce non-linearities in the calibration curve and affect long-term fidelity.

This study has shown that controlling the CSPR is not always sufficient to guarantee a constant metabolism throughout the cultivation (Figure 7). This, for example is visible in the cultivations with real-time CSPR setpoints of 0.04 and 0.05 nL/cell/day. Interestingly, it can be observed, within the context of each process, that the metabolism fluctuates more strongly at small iVVDs than at large ones (Figure 7, x-axis from left to right). This leads to the hypothesis that higher iVVDs result in larger volumes being exchanged from the beginning, leading to a more rapid shift in the metabolic state from the initial batch state to being driven by CSPR. Furthermore, our findings suggest that maintaining a constant CSPR, as seen in the case of iVVD 6.0 and PRI 1.7 vs. 2.0 where 1.7 exhibited a more constant CSPR and metabolic rates (Figure 4), can help to stabilize the metabolism. This is indicated by the narrow distribution of datapoints along the y-axis (Figure 7). Thus, our data reveal that the combination of constant CSPR profiles and iVVDs ≥9.0 allow to achieve constant specific metabolic rates of lactate and ammonia (<±25% fluctuation) (Figure 7). For cultivations with fixed daily VVDs, this was possible with a PRI of 1.7, which better matches the doubling time of this cell line, leading to constant CSPR profiles (also visible in Figure 4B). In literature, it has often been mentioned that a real-time CSPR controlled approach leads to a more constant metabolism or even a ‘steady-state‘ (Bergin et al., 2022; Rittershaus et al., 2022) and allows to maintain cell growth by preventing over- or under-supply of nutrients (in contrast to a CSPR setpoint over a certain time interval) (Müller et al., 2022; Rittershaus et al., 2022). These statements certainly hold true when N-1 process parameters are studied with unknown impact on growth and metabolism. However, our data highlight that for N-1 processes with known growth dynamics, the CSPR itself influences performance and metabolism independent of the perfusion rate control method.

### 4.3 N-1 perfusion performance - cell metabolism

The primary objective of the N-1 perfusion, like any other pre-culture step, is to generate enough biomass to inoculate the subsequent cultivation stage. However, the metabolic state of the cell culture also plays a crucial role, as it was reported to impact the N-stage performance and may require adaptations in the design of N-stage processes (Schulze et al., 2021; Stepper et al., 2020).

The PCA analysis based on specific rates reveals a metabolic adaptation in N-1 perfusion cultivations between day 0.5 and day 2.5 (Figure 2a). Day 2.5 was identified as a pivotal transition point based on clustering patterns observed in the PCA (Figure 2a) and correlation analysis of specific metabolic rates across timepoints (Figure 3). This transition marks a shift from initial batch conditions (day 0 – day 1) towards a CSPR driven metabolic state (Figures 3a–d, 4e,f). This metabolic shift observed around day 2.5 may partially reflect metabolic inertia during the initial phase of perfusion, where cells require time to adapt to the new dynamic environment, a phenomenon previously described in CHO cell cultures undergoing environmental transitions (Abu-Absi et al., 2013). Statistical criteria, including strong linear correlations (|r| > 0.8) from day 2.5 onwards (linear correlations including day 1.5 resulted in |r| < 0.5 for 12 out of 20 metabolites, see Figure 3) between daily CSPR and specific metabolic rates of 12 out of 20 analyzed metabolites (Figure 3), were used to define this transition in addition to the PCA clustering (Figure 2). Importantly, this adaptation point was consistently observed across all tested cultivation conditions, further supporting its significance in the metabolic behavior of the cell culture. In this context, also the publication of Stepper et al. (2020) was mentioned where phase dependent (transition after 48 h) yield coefficients were applied, which also points at a transition point in the same period of the cultivation (Stepper et al., 2020). To further validate the robustness of the PCA, we confirmed that clustering patterns and principal component loadings remained consistent when the analysis was repeated with and without imputed values (data not shown). This supports that the observed groupings are not artifacts of data preprocessing but reflect genuine biological variation. A CSPR driven metabolic state was also indicated by Schulze et al. (two different CSPR setpoints tested) and postulated by Konstantinov et al. who stated that all cells in a system with the same CSPR have a similar metabolic state (Konstantinov et al., 2006; Schulze et al., 2021). Besides the PCA analysis, a correlation analysis was performed. A strong linear correlation (|r| > 0.8) between the daily CSPR and the daily specific metabolic rates of 13 out of the 20 analyzed metabolites could be shown (Figure 3e). This correlation indicates that increasing the CSPR accelerates the overall metabolism. Similar trends were also observed for a different clone of the CHO-K1 GS cell line producing a distinct product, as shown in Supplementary Appendix Figures 7e, f, where the linear correlation between CSPR and specific metabolic rates of glucose and lactate was reproduced. This highlights the robustness and transferability of the metabolic behavior across cell lines. To ensure that the observed correlations were directly related to CSPR and not merely a co-correlation with cultivation time, we tested different CSPR profiles (downward, upward, and constant trend over time) by changing the PRIs and keeping the iVVD constant. As shown in Figures 4, 6, the metabolic rates are driven by the CSPR and are not a function of time or the specific CSPR profile. This indicates that the metabolic rates respond directly to the CSPR itself, regardless of whether the CSPR is increasing, decreasing, or remaining constant over time.

Interestingly, the daily specific metabolic rates of glucose, aspartate, glutamate, histidine, and tryptophan did not correlate with the daily CSPR (|r| < 0.4) (Figure 3e). This suggests that these metabolites may be regulated differently or may not be as sensitive to changes in CSPR. In the case of glucose, bolus additions were given which are not accounted for in the CSPR. These additions could impact specific glucose metabolic rates (Dowd et al., 2003). To accurately determine the effect on glucose, an experiment without bolus additions needs to be conducted.

We also observed no correlation between GLU and ASP specific metabolic rates and the CSPR. Given that these are structurally similar amino acids, it is plausible that a coherent mechanism explains why neither metabolic rate correlates with the CSPR. In the context of the BI CHO-K1 GS^−/−^ cell line used in this study, the media was not supplemented with GLN and only contains small concentrations of GLU. ASN, a key anaplerotic nutrient for GS-CHO cells (Val et al., 2023), is present in high concentrations. According to Zhang et al., a ratio of ASN/GLN favoring ASN is energetically preferred, and cells primarily use ASN to generate ASP and GLU (Zhang et al., 2016). Both explanations suggest that ASN might be used to produce GLU (via ASP and alpha ketoglutarate), in addition to GLN. Metabolic rates of GLU and ASP are therefore dependent predominantly on ASN consumption and despite the presence of ASP in the media, it is only consumed secondary to ASN, making a correlation with CSPR hard to determine. One hint to substantiate this hypothesis might be the increasing ammonia production with increasing CSPR, as the anaplerotic pathway of ASN is the predominant source of ammonia (Val et al., 2023). Another factor that may have influenced our results is the exclusive measurement of extracellular concentrations, not intracellular ones. It's possible that the metabolic rates of GLU and ASP do correlate with increasing CSPR, similar to ASN or GLN, but are immediately consumed intracellularly, which would not be detected with our current measurement methods. This could potentially explain the observed lack of correlation between the specific metabolic rates of ASP and GLU and the CSPR of perfusion for CHO cells. Future studies should consider measuring intracellular concentrations of these metabolites and employing Metabolic Flux Analysis (MFA) to provide a more comprehensive understanding of the metabolic and energetic activity of the cells. Despite not being directly influenced by changes in CSPR, the loadings plot of the PCA analysis suggests that aspartate and glutamate metabolic rates are the two major variables influencing PC2 (Supplementary Appendix Figure 1). This suggests that metabolic rates, even those not directly driven by the CSPR, still play a crucial role in the cell’s metabolism.

The primary challenge we faced in correlating the metabolic rate of TRP with the CSPR was most probably TRP’s high susceptibility to degradation in cell culture media which significantly complicates its precise measurement (Schnellbaecher et al., 2021). This factor can considerably alter the calculated metabolic rate of TRP, leading to potentially misleading outcomes. To address this issue, it’s necessary to accurately measure not only TRP but also its degradation by-products via LC-MS in future analyses. Additionally, metabolic rates of HIS were also not able to be correlated to CSPR. Histidine’s role, like ASP, is more tied to specific productivity, influencing protein synthesis and assembly, rather than cell growth (Ladiwala et al., 2023; Singh et al., 2024). In N-1 perfusion cultures, the focus is on increasing cell density and promote cell growth rather than the production of a recombinant protein. Hence, it is hypothesized that an increase in the CSPR does not result in an increase in the metabolic rate of histidine, explaining the lack of correlation.

As previously mentioned, the daily specific rates of several metabolites were found to correlate linearly with the daily CSPR. This denotes that the effect of the CSPR does not plateau in the CSPR range investigated in this study (0.035–0.113 nL/cell/day). Similar findings have been published by Dowd et al., who reported a plateau at CSPR rates of earliest 0.2 nL/cell/day, suggesting that the metabolism can be further accelerated (Dowd et al., 2003). Despite the CSPR’s influence on the cells’ metabolism, our data do not indicate a significant impact on the daily specific growth rate (Figure 6). This becomes especially evident when pointing at the cultivations with varied PRI (1.4, 1.7 and 2.0) and constant iVVD of 6 (Figure 4). Although the CSPR profiles showed opposite trends, which were followed by the lactate and ammonia specific rates, the specific growth rates remain comparable (Figure 6). This implies that while the CSPR alters the cells’ metabolism, it does not necessarily affect growth within a 24 h interval. This is further supported by our PCA analysis, which identified two clusters that point at distinct metabolic states throughout the process. Despite these metabolic states, the growth rate does not significantly influence principal component 1 and 2 (Supplementary Appendix Figure 1). This is evident from its position near the center of the two Principal Components in the PCA loadings plot.

## 5 Conclusion

This data-driven study aims to shed light into the leverage provided by the perfusion rates to optimize N-1 perfusion cultivations, displaying the interplay between parameters such as the iVVD, PRI and CSPR. The acquired information is essential to understand how to best impact cell growth and metabolism of these cultures, thereby developing efficient and scalable N-1 perfusion processes.

We have found that increasing the iVVD enhances cell growth, leading to higher final VCDs. However, the leverage provided by the iVVD is limited, as it seems to be stagnant at higher values. On a metabolic level, we have demonstrated that the CSPR drives the specific metabolic rates of many metabolites and amino acids after the metabolic adaptation on day 2.5. The linear correlations we observed between the daily CSPR and the daily specific metabolic rates indicate that increasing the CSPR accelerates the overall metabolism. However, such a correlation was not visible between the daily CSPR and the daily growth rate. This is because the different PRI we investigated did not influence the final VCD, denoting how cell growth is impacted by the overall medium exchange throughout the cultivation, rather than individual daily CSPRs. To further understand these observations, a metabolic flux analysis (MFA) specific to N-1 perfusion should be conducted for CHO-K1 GS^−/−^ cell lines. This would provide deeper insights into the metabolic pathways and mechanisms leading to these observations.

We have shown that both real-time CSPR control and fixed daily VVDs can be equally efficient strategies to drive cell growth at the N-1 stage. While both methods can be effective, our data suggests that real-time CSPR control does not necessarily provide a clear advantage over predefined rates in terms of enhancing cell proliferation. Additionally, real-time CSPR control does not necessarily offer benefits in terms of maintaining a more consistent metabolic state, even in scenarios where the iVVD is smaller. Therefore, fixed daily VVDs provide a viable alternative when real-time CSPR control is not feasible, such as when it is not implemented at production scale. Further studies might be necessary to validate these findings, and future investigations should aim to deepen our understanding of the observed effects. Our findings indicate a strong correlation between the CSPR and the specific metabolic rates of ammonia, lactate, and amino acids, suggesting that CSPR directly influences these metabolic rates (Figures 3e, 6). This was observed across a variety of cultivation conditions, including those with fixed daily VVDs and real-time CSPR control (Figure 7). Interestingly, we also observed a decrease in metabolic rate fluctuation with increasing iVVD, pointing towards a more constant metabolic state throughout the cultivation from day 2.5 onwards. This was true regardless of the perfusion control strategy used, although cultivations with more constant CSPR profiles demonstrated lower metabolic rate fluctuation.

From a production perspective, fixed rates define the media amounts more precisely. Despite the iVVD having the potential to be limiting in large-scale N-1 perfusion processes, there is still potential to influence the cellular metabolic state. As demonstrated in this paper, this can be achieved by using different CSPR profiles to induce different cellular metabolic states while reaching identical final VCDs at a set iVVD. Additionally, our data have shown that the CSPR profile not only affects metabolism but also viability, indicating that effects on the N-stage need to be investigated further. However, while this study provides valuable insights into optimizing N-1 perfusion processes at the bench scale, scaling these findings to industrial systems (≥2,000 L) presents unique challenges. As CSPR, with predefined perfusion rates, is an output parameter influenced by growth behavior, scaling requires careful adjustment of perfusion rates, particularly when predefined rates are used instead of online VCD-controlled adjustments. Additional challenges arise from hydrodynamic differences (e.g., shear stress) and mass transfer limitations, which might affect growth behaviour. Computational fluid dynamics (CFD) modeling and equipment characterization could help predict and mitigate these challenges. Future studies in pilot-scale systems will be essential to confirm scalability and ensure robust N-1 perfusion processes that maintain high cell densities and optimal metabolic states.

When utilizing probes for real-time monitoring and control, prior calibration is necessary, which increases process complexity. More data allows for better calibration, which however requires more preliminary experiments. Regulation could be based on cell permittivity, offering different avenues for process control and optimization. This is particularly useful during process development, as the optimal CSPR for growth is not known in advance, and fixed rates will always deviate from the setpoint when process parameters are investigated with unknown impact on growth dynamics. While it is possible to adjust the perfusion rate for the next interval based on offline data, this method is labor-intensive and prone to errors compared to sensor-based regulation, which is more precise.

While this study focused on the N-1 stage, which serves as a cell expansion phase rather than a production stage, to investigate cell growth and metabolism, we acknowledge that the impact of N-1 parameters on recombinant protein titer and CQAs during the production stage is critical. These aspects are the focus of a subsequent study, which builds on the findings presented here.

Overall, our findings highlight the importance of considering both the VCD and metabolic state in N-1 perfusion process design. Moving forward, it would be intriguing to explore the impacts on the production stage in subsequent experiments. This opens new opportunities for optimizing and scaling N-1 perfusion processes.

## Data Availability

The original contributions presented in the study are included in the article/Supplementary Material, further inquiries can be directed to the corresponding author.
