# Supplementary material for: Cell specific perfusion rates drive growth dynamics and metabolism in CHO N-1 perfusion processes independent of perfusion rate control method
Source: Front Bioeng Biotechnol. 2025 Jul 18;13:1608889. doi: 10.3389/fbioe.2025.1608889 (PMC12314661; doi:10.3389/fbioe.2025.1608889)
Supplement: Supplementary file 1 [file DataSheet1.docx]

**Appendix**





**Appendix Figure 1: Principal Component Analysis (PCA) Loadings plot.** Loadings of the first two principal components of the PCA depicted in Figure 2.

**
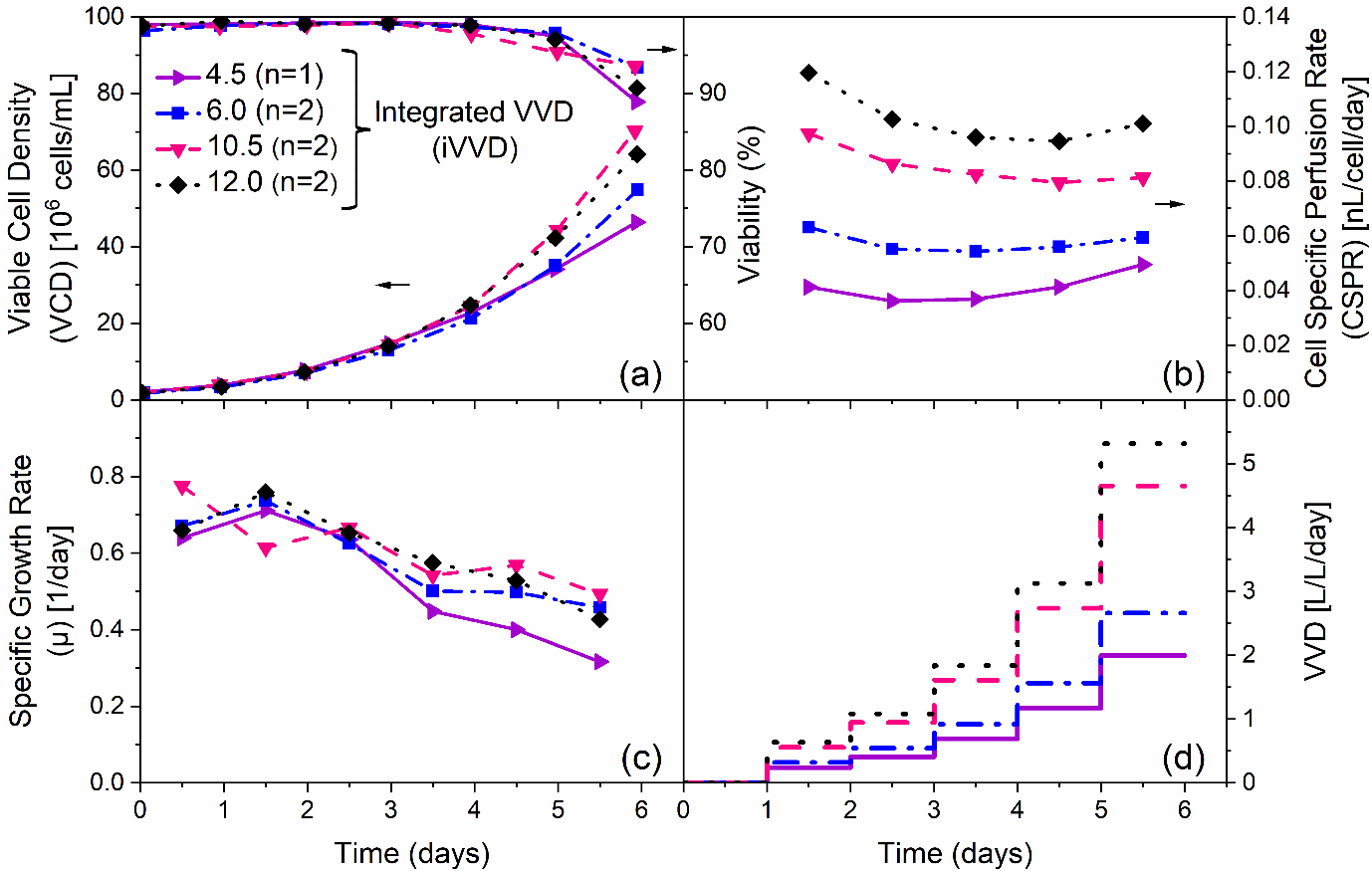
**

**Appendix Figure 2: Influence of the integrated VVD (iVVD) with perfusion rate increase (PRI) of 1.7 on process performance and cell specific perfusion rate (CSPR).** (a) Viable cell density (VCD) and viability, (b) CSPR, (c) specific growth rate (µ) and (d) pre-defined stepwise VVD profiles with a daily PRI of 1.7 over process time. The iVVD is the sum of the daily VVDs. n = number of cultivations. Mean values are depicted for n ≥ 2. Standard deviations are depicted by error bars for n ≥ 3.


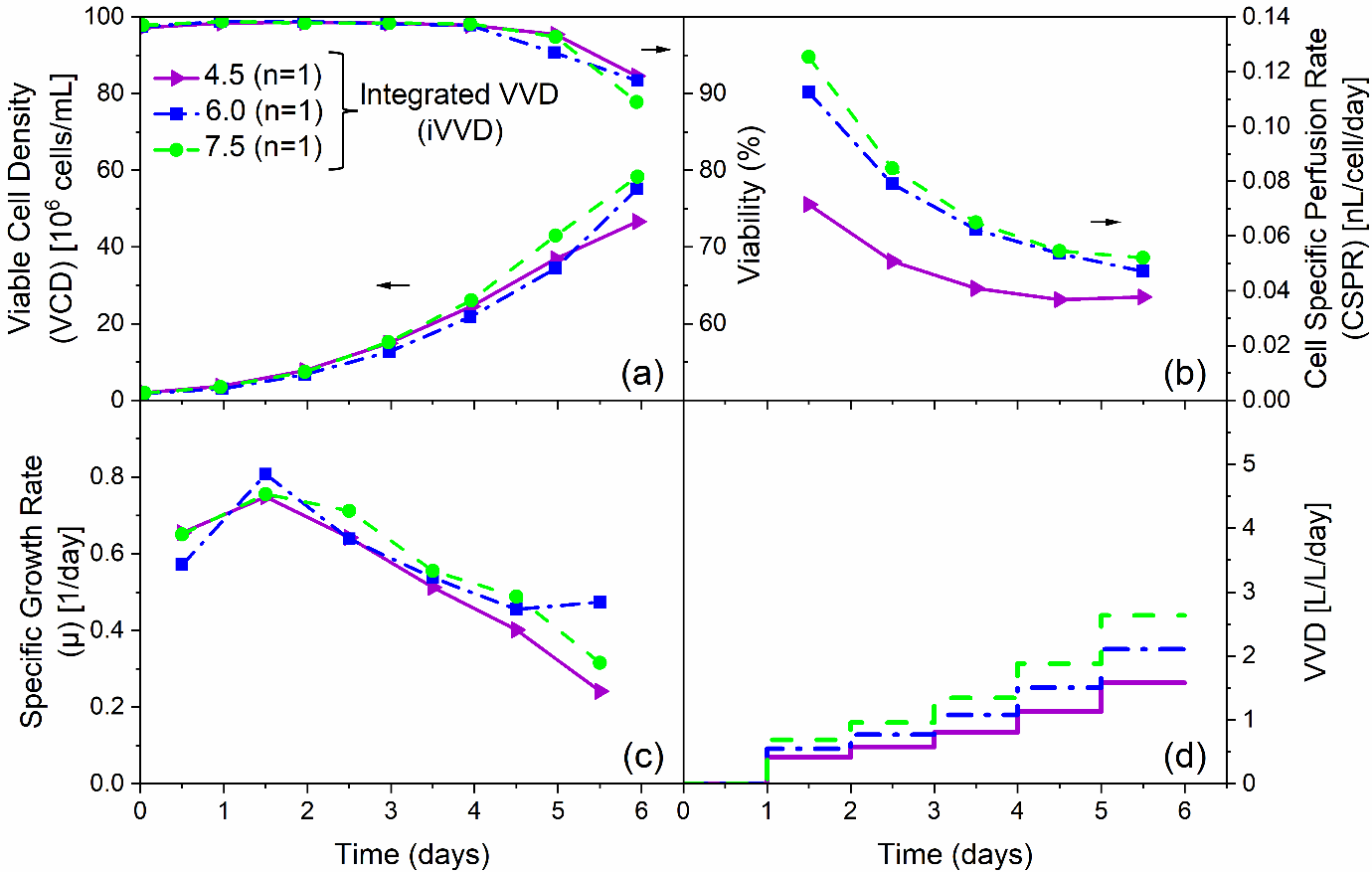


**Appendix Figure 3: Influence of the integrated VVD (iVVD) with perfusion rate increase (PRI) of 1.4 on process performance and cell specific perfusion rate (CSPR).** (a) Viable cell density (VCD) and viability, (b) CSPR, (c) specific growth rate (µ) and (d) pre-defined stepwise VVD profiles with a daily PRI of 1.4 over process time. The iVVD is the sum of the daily VVDs. n = number of cultivations.





**Appendix Figure 4: Calibration curves provided by the linear regression between permittivity and viable cell density (VCD).** (a) and (c) VCD over permittivity. (b) and (d) calibration residuals over VCD. Black squares depict data from cultivations used to create calibration curves. Magenta (a) & (b) and orange (c) & (d) circles represent values from the cultivations depicted in Figure 5. The calibration curves result from the linear regression of the calibration datapoints. The regression function, slope and R^2^ are provided.


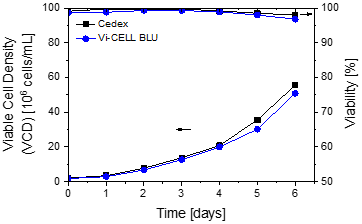


**Appendix Figure 5: Viable cell density (VCD) and viability over process time – comparison between Cedex Analyzer and Vi-CELL BLU cell count devices.** Measurements from the same offline samples. The depicted comparison was performed with the cell line used in this work.


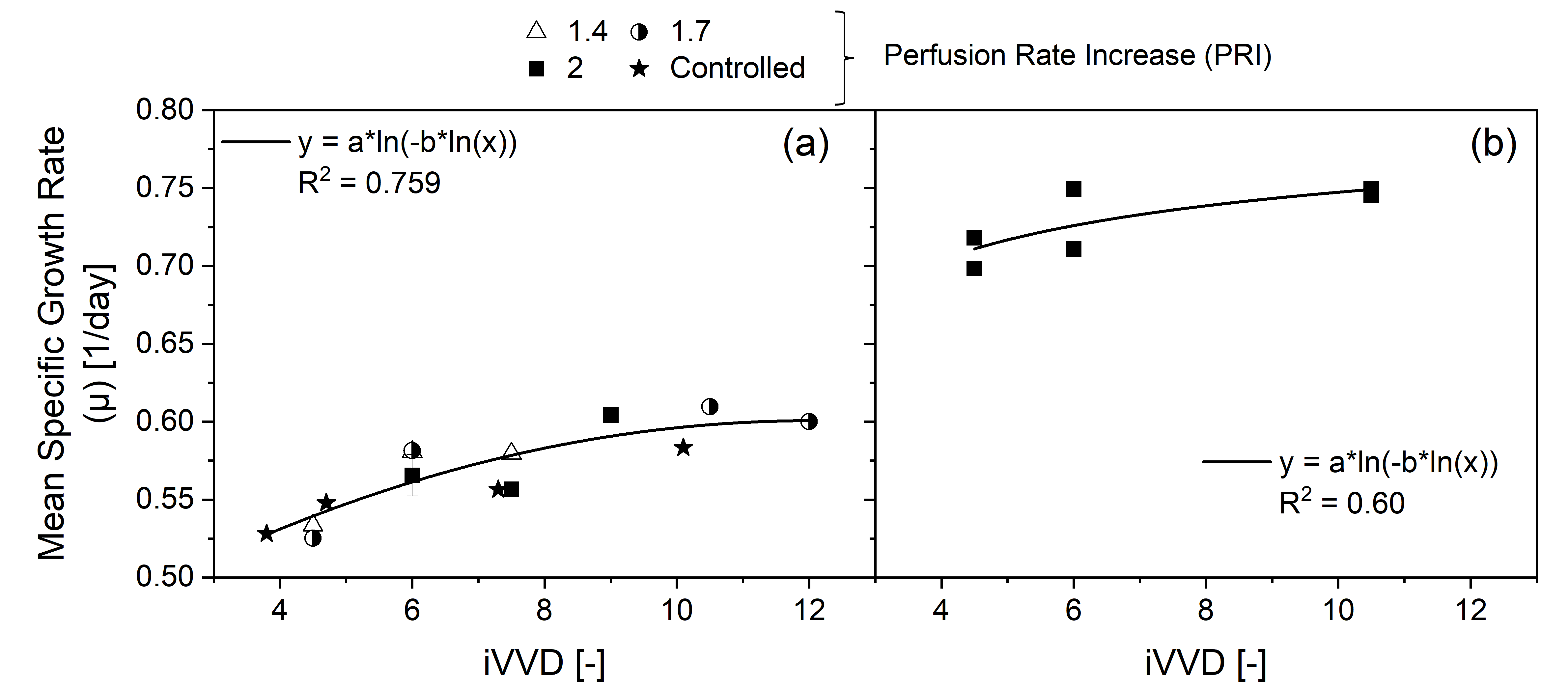


**Appendix Figure 6: Impact of the integrated VVD (iVVD) on the mean specific growth rate (µ) of each cultivation for two different clones of the CHO-K1 GS cell line.** The mean specific growth rate was chosen, rather than the final VCD, to compensate for any deviations in the seeding cell density. The iVVD was chosen, rather than the mean cell specific perfusion rate (CSPR), as it was either an input (fixed VVD cultivations) or a very accurately measured output (real-time CSPR control cultivations). (a) The cultivations in Figure 1 & 5 and Appendix Figure 2 & 3 are the source of the raw data. The logarithmic function (Bradley) used to describe the relationship and the R^2^ are provided. a=0.128 ± 0.0205, b=-44.632 ± 31.58. (b) The cultivations in Appendix Figure 7 are the source of the raw data. The logarithmic function (Bradley) used to describe the relationship and the R^2^ are provided. a=0.085 ± 0.0205, b=- 0720.075 ± 31.58.


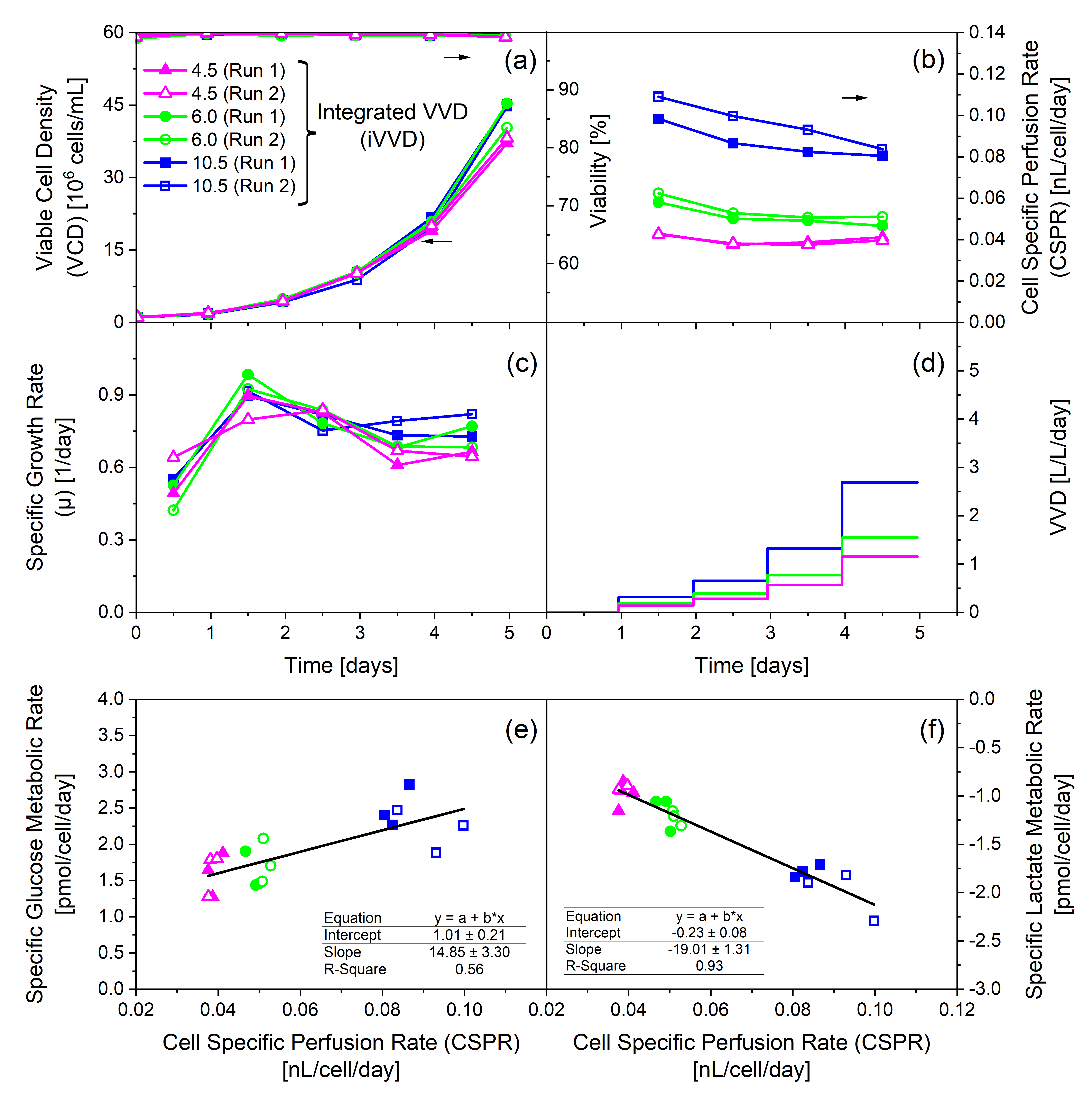


**Appendix Figure 7: Impact of integrated VVD (iVVD) on process performance and cellular metabolism on a different clone of the CHO-K1 GS cell line producing a distinct product.** Perfusion rate increase (PRI) was kept constant at 2.0. Cultivation conditions were similar as described under material and methods. Only difference was that due to overall higher specific growth the SCD was chosen with 1.0 x 10^6^ cells/mL. (a) Viable cell density (VCD) and viability profiles for cultivations with varying iVVD values (4.5, 6.0, and 10.5). (b) Cell-specific perfusion rate (CSPR) profiles for the same conditions. (c) Specific growth rate (µ) profiles for varying iVVD values. (d) Pre-defined stepwise VVD profiles with a daily PRI of 2.0. (e) Specific glucose metabolic rate and (f) specific lactate metabolic rate profiles over CSPR for cultivations with varying iVVD values (4.5, 6.0, and 10.5). Data demonstrate that trends observed for the original cell line are reproducible with a different clone of the CHO-K1 GS cell line producing a distinct product, confirming the linear correlation between CSPR and specific metabolic rates of glucose and lactate. These findings highlight the transferability of results across cell lines.
